# Single-cell lineage tracing with endogenous markers

**DOI:** 10.1007/s12551-024-01179-5

**Published:** 2024-02-07

**Authors:** Yan Xue, Zezhuo Su, Xinyi Lin, Mun Kay Ho, Ken H. O. Yu

**Affiliations:** 1 School of Biomedical Sciences, Li Ka Shing Faculty of Medicine, The University of Hong Kong, Pokfulam, Hong Kong SAR, China; 2 Laboratory of Data Discovery for Health Limited (D24H), Hong Kong Science Park, Units 1201-1206, 1223 & 1225, 12/F, Building 19W, 19 Science Park West Avenue, Hong Kong Science Park, Pak Shek Kok, New Territories, Hong Kong SAR, China; 3 Department of Orthopaedics and Traumatology, School of Clinical Medicine, Li Ka Shing Faculty of Medicine, The University of Hong Kong, Pokfulam, Hong Kong SAR, China

**Keywords:** Single-cell lineage tracing, Somatic nuclear mutations, Mitochondrial mutations, Spatial clonal evolution

## Abstract

Resolving lineage relationships between cells in an organism provides key insights into the fate of individual cells and drives a fundamental understanding of the process of development and disease. A recent rapid increase in experimental and computational advances for detecting naturally occurring somatic nuclear and mitochondrial mutation at single-cell resolution has expanded lineage tracing from model organisms to humans. This review discusses the advantages and challenges of experimental and computational techniques for cell lineage tracing using somatic mutation as endogenous DNA barcodes to decipher the relationships between cells during development and tumour evolution. We outlook the advantages of spatial clonal evolution analysis and single-cell lineage tracing using endogenous genetic markers.

Various fundamental biomedical questions in development and disease can be addressed by integrating genotype and phenotype information from single cells, which requires simultaneous measurement of the lineage relationship and cell fates. Coupling lineage tracing with trajectory inference helps disentangle complex state transitions. Though most approaches for cell lineage tracking rely on engineered genetic barcodes labelling individual cell states with high resolution, these methods are confined to the model organisms. In humans, lineage tracing has depended on naturally occurring somatic nuclear and mitochondria genome mutations arising from the random errors of DNA replication, DNA repair, or random integration of transposable elements in the genome. These somatic mutations are permanent and transmitted to the progeny and, therefore, serve as endogenous barcodes mainly consisting of copy number variants (CNVs), single-nucleotide variants (SNVs), microsatellites repeat, L1 retrotransposition elements and mtDNA mutations (Baron and van Oudenaarden 2019; Ludwig et al. 2019; Woodworth et al. 2017). Here, we reviewed the advancements of endogenous markers-enabled single-cell lineage tracing, the major characteristics and challenges of computational algorithms for single-cell variant calling, as well as outlooked the advantages of spatial clonal evolution analysis and single-cell lineage tracing using endogenous genetic markers.

## Somatic nuclear mutations enabled lineage tracing

**Fig. 1:**
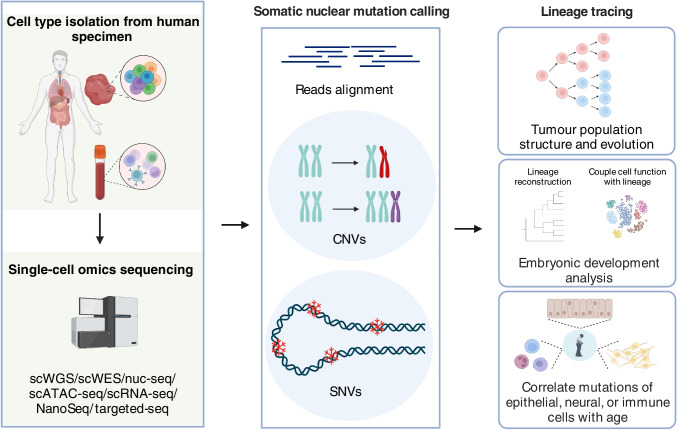
Somatic nuclear mutation enabled lineage tracing. Advances in single cell sequencing pave the way to infer cell lineage information by harnessing somatic mutations, typically CNVs or SNVs, as naturally occurring genetic markers. Tumour population structure and evolution, post-zygotic events of human embryos, and mutational landscape in various cell types are illustrated by tracking clonal dynamics

Somatic cells acquire mutations throughout the lifetime of an individual (Lodato et al. 2015). To use somatic mutations as endogenous markers for lineage tracing, it is essential to capture shared mutations between multiple cells from the same individual. Advances in single-cell sequencing pave the way to infer cell lineage information by harnessing somatic mutations, typically CNVs or SNVs, as naturally occurring genetic markers (Fig. 1).

### Tracing tumour evolution with somatic nuclear mutations

Subchromosomal somatic CNVs are stretches of DNA more than 1 kilobase (kb) long present in different copy numbers when compared with the reference genome (Baron and van Oudenaarden 2019). CNVs can be detected in both healthy and disease tissues from single-cell sequencing data including those with low coverage and thus are potentially useful for the lineage tracing (Abyzov et al. 2012; Cai et al. 2014; Knouse et al. 2016; McConnell et al. 2013). Tumour population structure and evolution were illustrated by CNVs from single-nucleus sequencing in human breast cancers (Navin et al. 2011). Polygenomic tumour analysis revealed three distinct clonal subpopulations that probably represent sequential clonal expansions, and analysis of the monogenomic primary tumour and its liver metastasis indicated that a single clonal expansion formed the primary tumour and seeded the metastasis. The development of nuc-seq allowed for highly confident CNVs profiling and showed point mutations evolved gradually, generating extensive clonal diversity in breast cancer (Wang et al. 2014). Moreover, topographic single-cell sequencing developed was able to measure CNVs of single tumour cells while preserving their spatial context (Casasent et al. 2018). This study revealed that one or more clones escape the ducts and migrate into the adjacent tissues to establish invasive ductal carcinoma. Studies have tried to leverage CNV inference from scRNA-seq to interrogate genetic clonality regardless of low resolution and noise background (Kurtenbach et al. 2021; Zhou et al. 2020). In a comprehensive single-cell atlas of gastric cancer, inter- and intrapatient lineage similarities and differences were identified by CNVs among 34 distinct cell-lineage states in 48 samples from 31 patients across clinical stages and histologic subtypes (Kumar et al. 2022). Multiple lineage tracing integrated with CNV states, SNV states and viral lineage barcoding using a colon cancer organoid model over 100 generations allowed the construction of highly detailed evolutionary trees and demonstrated sequential loss of chromosomes 18 and 4 correlates with fitness advantage in tumour evolution (Kester et al. 2022). The high prevalence of CNV changes in blood malignancies permits unambiguous identification of the clonal lineage of cell populations within heterogeneous phenotypes and facilitates the tracking of the trajectories of malignant and of immune cell populations in acute myeloid leukaemia and chronic lymphocytic leukaemia (Penter et al. 2021, 2022).

### Tracing embryonic development with somatic nuclear mutations

SNVs are frequent variations in a single nucleotide and represent particularly promising endogenous lineage markers due to their high abundance and frequent neutral function. Single-cell DNA sequencing of human embryonic cells enables detailed examination of the timing and mutational profile of post-zygotic events. Bizzotto et al. quantified the mosaic fractions (MF, fractions of cells carrying the variant) in early cell generations of progenitors by sequencing five high-depth bulk whole-genome sequencing (WGS) samples (2 brain, 1 heart, 1 spleen, 1 liver) of three individuals, calling somatic single-nucleotide variants (sSNVs) and constructing lineage trees based on these variants (Bizzotto et al. 2021). In one of the individuals, previous data of 20 single neurons WGS from the same group resolved 82/297 sSNVs into clones, tracing each mutation back to its origin. It was found that the change in MF across cell generations was asymmetrical and much slower than the expected two-fold reduction per cell division. Moreover, an MF reduction below around 0.6% shows sSNVs limited to one or two germ layers and indicates the start of gastrulation and organogenesis. Furthermore, brain-specific sSNVs showed relatively high MF in the forebrain, and the number of these progenitor cells is estimated to be 50–100. Chapman et al. characterised the phylogeny trees of foetus blood development using WGS of hundreds of single-cell-derived haematopoietic colonies along with various tissues of known embryonic origin from healthy 8 and 18 post-conception weeks (pcw) foetuses (Spencer Chapman et al. 2021). From mutations in haematopoietic stem and progenitor cells (HSPCs) that were shared with the gut epithelium, it was discovered that more than 60 lineages give rise to gut epithelium and blood HSPCs. In the characterisation of other tissues, it was found that during the 4–16-cells stage trophoblast diverges from the blood precursors, which have five detected lineages.

### Somatic nuclear mutations in normal tissues

The single-cell WGS approach can also be used to inspect human adult epithelial cells and correlate the extent of mutations in a cell type with participant age. Huang et al. studied the somatic mutations landscape in normal human proximal bronchial basal cells (PBBCs), which are the likely progenitor cells for squamous cell carcinoma (Huang et al. 2022). Single-cell WGS of normal human bronchial epithelia from 14 never-smokers (aged 11–84) and from 19 smokers (aged 44–81) were sequenced and profiled. It was observed that the number of mutations in PBBCs increased linearly with age in never-smokers, while in smokers, mutations also increased linearly, but at a significantly higher rate. In the PBBCs, no statistical enrichment was found for lung cancer or pan-cancer driver gene mutations. Brazhnik et al. used single-cell WGS to characterise the mutational landscape of differentiated human liver hepatocytes compared to adult liver stem cells (LSCs) (Brazhnik et al. 2020). It was uncovered that differentiated hepatocytes have significantly higher spontaneous mutational frequencies (that also increased with age) compared to LSCs. For young individuals only, LSC clones were establishable and showed consistency in SNV between parent clones and kindred single cells. Further, mutational signatures of LSCs and young hepatocytes were similarly L2 dominated, while the signature of aged hepatocytes differed and was L1 dominated.

Others have yet again used single-cell WGS to investigate human neural or immune cell types as a function of age. Lodato et al. (2015) identified thousands of sSNVs by single-neuron WGS of 36 neurons. The detected somatic mutations are shared between multiple neurons and demonstrate lineage relationships and signatures of mutagenic processes, such as transcription-associated DNA damage and a preponderance of meC > T deamination. They demonstrated that somatic mutations can be used to reconstruct the developmental lineage of neurons, suggesting a potential “population genetics” of brain cells. sSNVs in coding regions of genes involved in nervous system development and mature neuronal function show that the very genes used for the function of a neuron were those most likely to be damaged during its life. Three years later, Lodato et al. identified genome-wide sSNVs in 159 single neurons from the prefrontal cortex and hippocampus of 15 healthy individuals (aged 0.33–82 years) and 9 individuals with early-onset neurodegeneration and found that sSNVs mainly increased linearly with age in both parts of the brain, but with a significantly higher rate in the hippocampus, and that sSNVs were notably more abundant in neurodegenerative disease (Lodato et al. 2018). Zhang et al. performed genome-wide mutation calling in human B lymphocytes from newborns to centenarians (Zhang et al. 2019) and found that somatic mutations increase with age, with less than 500 mutations per cell in newborns and over 3000 mutations per cell in centenarians. Moreover, mutation signatures in the normal B cells were found to be consistent with those previously identified in B cell leukaemia, suggesting that age is the major risk factor for some cancers. Abascal et al. developed a new WGS library preparation approach, NanoSeq, which solves the end-repair errors introduced by BotSeqS (Abascal et al. 2021). NanoSeq was used to compare mutation rates in stem cells and terminally differentiated cells. Although it was hypothesised that differentiated cells have higher mutation rates, this study showed that granulocytes have a similar mutation burden as haematopoietic stem and multipotent progenitor cells (HSC/MPPs). The mutation landscape and signatures in neurons and smooth muscle cells were also examined, with neurons having more T > C substitutions at ApT sites and indels than other tissues. For smooth muscle cells, the extent of mutations and indels increases linearly with age.

Despite somatic CNVs and SNVs representing a rich resource for lineage tracing mutations, the limitation of their application is also obvious. The readout of nuclear somatic mutations heavily relies on deep sequencing of the whole genome or exome of single cells which cannot be applied at scale due to substantial error rate and cost (Baron and van Oudenaarden 2019; Bizzotto et al. 2021; Ludwig et al. 2019; Spencer Chapman et al. 2021; Woodworth et al. 2017). The future use of deeper or targeted sequencing approaches is anticipated to improve our ability to identify more somatic nuclear mutations at a large scale to build lineage history.

## Somatic mitochondrial mutation enabled lineage tracing

In recent years, mitochondrial DNA (mtDNA) has been recognised as a natural genetic marker in clone and lineage tracing of native health and disease human cells to relate clonal dynamics with gene expression and chromatin accessibility (Lareau et al. 2021; Ludwig et al. 2019; Penter et al. 2021; Xu et al. 2019). Even without prior knowledge of nuclear mutations, due to the high levels of mutation rate, copy number, and heteroplasmy, mitochondrial mutations can confidently resolve clonality in primary human cells, allowing for quantitative analysis of gene expression. Moreover, the cost-effective sequencing of the small mitochondrial genome favours the broad application of mitochondrial mutation barcoding for clonal charting during embryonic development, stem cell differentiation and disease progression in humans.

### MtscATAC-seq: parallel profiling of mitochondrial DNA genotype and epigenomic variability

**Fig. 2:**
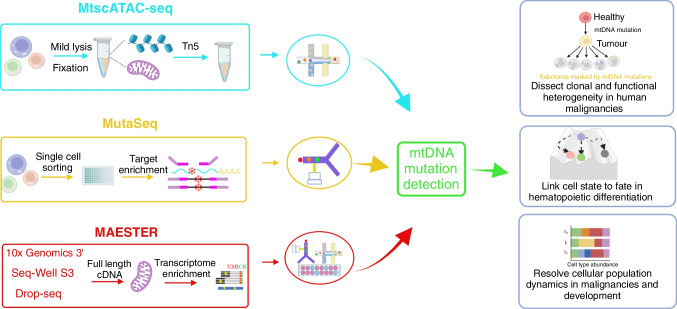
Somatic mitochondrial mutation enabled lineage tracing. mtscATAC-seq processes whole cells to retain mtDNA and improve genome coverage by mild cell lysis or permeabilization required for the Tn5 enzyme to integrate adapters into accessible nuclear chromatin and mtDNA. mtscATAC-seq simultaneously infers mtDNA heteroplasmy, clonal relationships, cell state, and accessible chromatin variation in individual cells. MutaSeq increases the average coverage of genomic and mitochondrial mutations by targeting mutation sites of interest during cDNA amplification and enabled lineage tracing with the combination of single-cell transcriptomics and mitochondrial somatic variants. MAESTER (mitochondrial alteration enrichment from single-cell transcriptomes to establish relatedness) enriches all mitochondrial transcripts in full-length cDNA transcript yields from the most commonly used high-throughput scRNA-seq platforms, such as the 10 × Genomics 3′ protocols, Seq-Well S3 and Drop-seq, to resolve clonal relationships

Although single-cell assay for transposase-accessible chromatin using sequencing (scATAC-seq) can profile accessible chromatin in thousands of cells per experiment, it relies on nuclei processing, which depletes cytoplasmic mitochondria. Thus, mitochondrial single-cell assay for transposase-accessible chromatin with sequencing (mtscATAC-seq) was developed based on the droplet-based scATAC-seq techniques to simultaneously infer mtDNA heteroplasmy, clonal relationships, cell state and accessible chromatin variation in individual cells (Lareau et al. 2021). mtscATAC-seq processes whole cells to retain mtDNA and improve genome coverage by mild cell lysis or permeabilisation required for the Tn5 enzyme to integrate adapters into accessible nuclear chromatin and mtDNA (Fig. 2). The results obtained by leveraging somatic mtDNA variation indicate that naturally occurring genetic mtDNA barcodes have the potential to resolve clonal heterogeneity within malignancies and assess clonal dynamics in hematopoiesis, while also providing rich information on cell state variation. This multi-omic massively parallel protocol enhances our understanding of mtDNA genotype–phenotype correlations and reconstructs clonal dynamics across diverse areas of human health and disease.

In the context of mitochondrial disease, with the application of mtscATAC-seq in thousands of blood cells from unrelated patients, mtDNA heteroplasmy and segregation dynamics of pathogenic mutations in each blood-cell lineage that affect the function of immune cell types were dissected (Walker et al. 2020). The study observed a broad range of A3243G heteroplasmy across all cell types, yet the T cell lineage had a significant reduction in heteroplasmy when compared to peripheral blood mononuclear cells, which consist of multiple cell types originating from a common stem and progenitor pool, indicating purifying selection within the T cell lineage. Thus, exploiting endogenous mechanisms that survey and purify pathogenic mtDNA alleles may inspire novel therapeutic approaches for mitochondrial diseases.

### Muta-seq: simultaneous mapping of mitochondrial and genomic mutations

With the combination of single-cell transcriptomics and mitochondrial somatic variants enabled lineage tracing, MutaSeq effectively distinguished between cancer stem cells and progenitor cell populations in acute myeloid leukaemia patients (Velten et al. 2021). Targeting mutation sites of interest during cDNA amplification (other than reverse transcription) will increase the average coverage of genomic and mitochondrial mutations without the formation of undesired by-products, hence charting the capabilities of MutaSeq in differentiating and mapping the molecular implications of oncogenic mutations (Fig. 2). More importantly, this mitochondrial mutation-enabled clonal tracking method can be applied to distinguish between healthy and cancerous clones in various cancer types and improve our understanding of cancer progression and relapse. However, due to the high cost and low throughput of the modified Smart-seq2 sequencing technology, the use of mtDNA as a natural genetic cell barcode for clonal tracking is limited. Requiring prior knowledge about a certain disease is another barrier to the wide application of MutaSeq.

### MAESTER: expansion of mitochondrial mutation detection to high throughput nature

MAESTER (mitochondrial alteration enrichment from single-cell transcriptomes to establish relatedness) was developed to overcome these limitations (Miller et al. 2022a). MAESTER enriches all mitochondrial transcripts in full-length cDNA transcript yields from the most commonly used high-throughput scRNA-seq platforms, such as the 10 × Genomics 3′ protocols, Seq-Well S3 and Drop-seq, to achieve full-length coverage of the mitochondrial genome while preserving cell-identifying barcodes (Fig. 2). The approach is ideal for determining clonal relationships between more divergent subsets of cells. The introduction of MAESTER broadens the use of naturally occurring barcodes generated by mtDNA alterations to enable discoveries in human biology.

In acknowledging the power and broad applicability of mtDNA lineage tracing, it is important to also be aware of its limitations. In tissues and cells that are in the early stages of development, such as embryos and young animals, where the extent of mitochondrial mutations is insufficient for clonality analysis, this lineage tracing technique is not suitable. Furthermore, seeing as selective mitochondrial inheritance or intercellular mitochondria transfer may affect the accuracy of mtDNA lineage tracing, a nuclear DNA tracing method should be utilised to track clone dynamics parallelly.

## Common principles, characteristics, and choice of nuclear and mitochondrial mutation for somatic lineage tracing

CNVs show promise as somatic lineage tracing markers because they can be identified from low-coverage sequencing, making it cost-effective to sequence many single cells for variant discovery. SNVs are a major source of evolutionary and disease-causing mutations, but they can also occur frequently in non-coding regions without functional effects on somatic cells. Therefore, somatic SNVs are abundant and likely to be functionally neutral, making them useful as lineage markers. The precise rates of somatic SNV mutation vary between species and methodologies used in the studies. However, the development of new algorithms for single-cell genome sequencing data interpretation and expanding the range of cell types and tissues subjected to sequencing can help resolve these discrepancies. Retroelement transposons, particularly L1, are present in large numbers in the genome and have the ability to move to new genomic sites during cell division. The human brain has been extensively studied for clone identification and lineage reconstruction using L1 elements. However, the reported number of somatic L1 mobilisation events in the brain varies across different studies mainly subject to the different detection methods employed (Baillie et al. 2011; Coufal et al. 2009; Evrony et al. 2012, 2016; Upton et al. 2015). LINE-1 elements were combined with SNVs to create a more precise lineage map (Evrony et al. 2015), demonstrating the potential to combine different naturally occurring mutations for retrospective lineage tracing.

The frequency of naturally occurring mutations varies in different tissues and along the life span. In the human brain, less than 2% of neurons were found to exhibit aneuploidy, while 41% of neurons harboured a few megabase-scale CNVs (Evrony et al. 2015; McConnell et al. 2013). In contrast to mature cells, early preimplantation human and macaque embryos have shown remarkably high rates of aneuploidy and CNVs. Single-cell microarray profiling of preimplantation human embryos and scDNA-seq of macaque embryos have revealed that 74% of embryos have at least one blastomere with abnormal chromosome copy numbers (Daughtry et al. 2019; Vanneste et al. 2009). Additional studies indicate that there is a relatively similar burden of somatic SNVs per cell at birth across different cell types. However, there is a significant increase in SNV accumulation with age, albeit at different rates in different cell types. These studies have shown that fibroblasts from a human toddler have approximately 900 somatic SNVs per cell; B lymphocytes in newborns have around 460 SNVs, which increase to approximately 3000 in centenarians; and newborn hepatocytes have approximately 1000 SNVs, increasing to 4000–5000 in elderly individuals (Brazhnik et al. 2020; Dong et al. 2017; L. Zhang et al. 2019). Interestingly, most of these SNVs are unique to individual cells within the sample, and computational analysis has revealed that they can be attributed to an ageing clock-like mutational process.

Recent studies further demonstrated the mutation diversity of mtDNA across human tissues (M. Li et al. 2015; Ye et al. 2014). Through the analysis of mitochondrial genotypes from 8820 individual samples across 49 tissues, it was observed that there is significant variation in the proportion of mitochondrial reads mapping to the mitochondrial transcriptome across different tissues and this variation is consistent with the known differences in the absolute numbers of mitochondria and the levels of mitochondrial gene expression in each tissue (Ludwig et al. 2019). This suggests the broader applicability of mtDNA variants as natural genetic markers for clonal tracking. The development of high-throughput mitochondrial variant enrichment platform (Miller et al. 2022b) and mitochondrial variant calling pipelines (Kwok et al. 2022) even significantly expand the application of mitochondrial variant-based lineage tracing.

Retrospective lineage tracing involves analysing the genomes of single cells or small groups of cells, which requires amplification of DNA to generate enough material for next-generation sequencing. However, the amplification process is error-prone and can introduce sequence or structural errors that lead to false-positive mosaic structural variants, microsatellite variability, and SNVs (Baron and van Oudenaarden 2019; Woodworth et al. 2017). Uneven amplification across the genome can also cause false-positive CNV calls and false-negative sequence calls due to allelic dropout. To decrease the technical artefacts, one broad class of whole-genome amplification strategies is amplifying the genome in vitro using highly processive DNA polymerases, degenerate oligonucleotide priming PCR, multiple annealing and looping-based amplification cycles, or multiple displacement amplification (MDA) (Dean et al. 2002; Evrony et al. 2015; Fu et al. 2015; Lodato et al. 2015; Sidore et al. 2016; J. Wang et al. 2012). Fragmenting the genome into small pieces and amplifying with random priming could amplify a more even genome than MDA and is thus particularly well-suited for CNV study (Cai et al. 2014; Navin et al. 2011). Another strategy to avoid the technical artefacts is amplifying the genome in vivo, in cells or whole organisms, by cloning and cell culture (Behjati et al. 2014; Leung et al. 2016; Y. Wang et al. 2014). Therefore, it is crucial to consider the frequency and types of errors introduced and choose an approach that balances signal and noise for the specific experiment.

When designing a somatic mutation-based lineage tracing experiment, the strengths and weaknesses of both nuclear and mitochondrial mutations need to be considered. Nuclear mutations occur in the DNA located in the nucleus of the cell and are subject to somatic mutations during cell division. The analysis of nuclear mutations requires amplification of DNA from single cells, which can introduce errors during the amplification process. Mitochondrial mutations occur in the DNA located in the mitochondria of the cell and are also subject to somatic mutations. However, mitochondrial mutations occur at a higher frequency than nuclear mutations, and their abundance can vary among cells due to differences in mitochondrial DNA copy number (Stewart and Chinnery 2015a). Mitochondrial mutations can be used to infer lineage relationships, but their interpretation can be more challenging due to the potential for heteroplasmy, which refers to the co-existence of multiple mitochondrial genomes within a single cell (M. Li et al. 2015; Ludwig et al. 2019; Wallace and Chalkia 2013). Overall, the choice of whether to use nuclear or mitochondrial mutations for somatic mutation-based lineage tracing depends on the specific research question and the available tools or techniques. In some cases, a combination of both nuclear and mitochondrial mutations may be necessary to gain a comprehensive understanding of cell lineage dynamics and their functional implications.

## Bioinformatics pipelines for somatic mutations lineage tracing

Tracing cellular lineage through somatic and mitochondrial DNA mutations involves different steps. These can be summarised as sample collection and preparation, sequencing, data processing, variant calling, clonal inference and data interpretation. The development of next-generation sequencing (NGS) has played a pivotal role, offering detailed genetic information about whole-genome, exome or targeted regions depending on the scope of the study. Various data processing methods have been developed to discriminate true mutations from sequencing artefacts, and somatic mutations are selected for further biological interpretation. Clone inference utilises these mutations to define subclones of cells with different genotypes. These clones can sometimes be mapped to the evolutionary trajectories of cells and help to decipher the roles of important mutations (Miles et al. 2020). The final interpretative phase involves contextualising the mutational data within biological systems, aiming to understand functional consequences, evolutionary history or clinical implications. Among all these steps, proper computational tools play a vital role in variant calling and clonality inference.

### Bioinformatics pipelines for variant calling

Detecting somatic SNVs, indels, CNVs or structural variants has become a typical application since the development of sequencing data. Yet, due to the novelty of the lineage tracing field using single-cell data, there is no consensus upon a gold standard pipeline for variant inference purposes, and ongoing development of methodologies is observed. In lieu of this, we set out to perform a fair comparison between various somatic mutation and structural variant detection algorithms from their principles to application data types in Table 1. Mutect2 (Benjamin et al. 2019) is part of the GATK suite of tools and is designed for calling somatic mutations in cancer genome data based on DNA sequencing data. It calculates a likelihood for somatic genotyping and detects somatic mutation using the aligned reads files of tumour and normal paired samples and is also used in RNA sequencing data. CTAT (Fangal 2020) uses tree-based classification for variant filtering and is specifically designed for RNA sequencing data. VarScan2 (Koboldt et al. 2012) and Strelka2 (Kim et al. 2018) are two other tools which are popular in detecting both somatic and germline mutations. VarScan2 uses a Fisher’s exact test for positive detection, while Strelka2 uses final empirical variant scoring and can handle data from a variety of sequencing technologies.

**Table 1:** Comparison of somatic SNV/indel and structural variant detection algorithms

Method	Variant detection model	Input	Output	Data type
Mutect2	Pair-HMM probabilistic model for sequence alignment to assign a likelihood for somatic genotyping	1) The reference FASTA file to which reads were aligned 2) Bam file of tumour sample 3) Bam file of normal sample (optional)	VCF file (somatic mutation)	DNA and RNA sequencing data
VarScan2	Fisher’s exact test for positive detection; a false-positive filter based on nine empirically derived criteria	1) The reference FASTA file to which reads were aligned 2) Sorted BAM files of samples	VCF file (somatic mutation, loss of heterozygosity and CNVs)	Exome sequencing data
Strelka2	Mixture binomial model (germline analysis)/final empirical variant scoring (EVS)	1) BAM or CRAM files of tumour/normal sample pairs 2) Candidate and/or forced-call alleles from VCF (optional)	VCF file (germline mutation and somatic indels)	Single-cell exome sequencing data
MuSE	F81 Markov substitution model which provides the estimation of alleles frequency; a two-component Gaussian mixture model for further filtering	1) the reference FASTA file to which reads were aligned 2) BAM files of tumor/normal sample pairs 3) the dbSNP variant call format (VCF) file that should be bgzip compressed, tabix indexed and based on the same reference genome as (1)	VCF file (list of somatic variants)	WGS data
CTAT	GATK Best Practices pipeline (Mutect2) for variant calling; tree-based classification for variant filtering	Sequencing reads file (FASTQ files)	Summary reports and visualizations	RNA sequencing data
FreeBayes	A dynamic windowing approach to assemble haplotype observations; using Bayesian framework to establish a maximum a posteriori estimate of the genotype; using marginal likelihoods for individual genotypes	1) The reference FASTA file to which reads were aligned 2) Sorted BAM files of samples	VCF file (SNPs, INDELs, and haplotype variants)	short read sequencing data
cellsnp-lite	SNPs are filtered based on their allele frequencies and coverage defined by users	1) Aligned reads file (bam/sam/cram) 2) Candidate and/or forced-call alleles from VCF (optional)	1) UMIs (if exist) or reads for all A, C, G, T and N bases 2) SNPs’ position, original allele and alternative allele	RNA sequencing data (bulk or single cell)
SCmut	Using samtools and varscan2 to detect somatic mutation, then detecting cell level mutation	1) BAM file of scRNA-seq data 2) BAM file of bulk-cell DNA-sequencing (e.g. whole exome sequencing—WES) of matched samples (tumour and normal), or a list of somatic mutation	Mutation information at single cell level	scRNA-seq data

Except for somatic mutations, other structural variants also play an important role in cancer development and provide important information for lineage tracing. MuSE (Fan et al. 2016) and FreeBayes (Garrison and Marth 2012) are designed for the detection of structural variants, including deletions, insertions, inversions and translocations, in next-generation sequencing data. Meanwhile, cellsnp-lite (X. Huang and Huang 2021) enables mutation detection not only for bulk RNA sequencing data but also at single cell level when using scRNA-seq. It is implemented in C/C +  + with computational parallelisation design to handle the high time cost problem raised by large single-cell data size. It also takes into account the unique features of single cell data, such as low coverage and high noise levels. SCmut (Vu et al. 2019) utilises the somatic mutation detection results from Mutect2 to detect somatic mutations at single cell level.

### Computational strategies for clonality inference

In parallel with the emergence of detecting mutations, different computational strategies were developed to infer clonality from various single-cell sequencing techniques. The MEDALT (Minimal Event Distance Aneuploidy Lineage Tree) algorithm infers the evolution history of a cell population based on single-cell copy number profiles and lineage speciation analysis (F. Wang et al. 2021). In the context of breast cancer, MEDALT effectively prioritises genes that are essential for cancer cell fitness and predict patient survival, demonstrating higher accuracy compared with phylogenetics approaches in reconstructing copy number lineage. EMBLEM (Epigenome and Mitochondrial Barcode of Lineage from Endogenous Mutations) (Xu et al. 2019) and mgatk (Mitochondrial Genome Analysis Toolkit) (Lareau et al. 2021) were developed as methods to track cell lineage in ATAC-seq and mtscATAC-seq data using endogenous mitochondrial DNA variants. Based on mtDNA variants, EMBLEM infers cell lineage and overlays the epigenomic clonotype information. In EMBLEM, after read alignment, filtering steps include the removal of reads with poor mapping quality and PCT artefacts, as well as accounting for mitochondrial heteroplasmy and sequencing errors, such as a minimum read count from both forward and reverse reads to avoid strand imbalance and flagging variant allele frequency (VAF) over 0.9 as homoplasmic variants.

Considering that the mtDNA copy number in each cell varies and informative clonal mutations can occur at very low allele frequencies, mgatk was constructed to be largely independent of the mean allele frequency and robust to variability in the genomic ploidy of a cell. Applying Pearson correlation coefficient between allele counts for cells with alternate alleles, mgatk improves strand concordance and reduces the effects of photobleaching of sequencing. A threshold variance mean ratio (VMR) filter is used to output cells with matching mutations in both allele strands. Both EMBLEM and mgatk generate single-cell lineage information and a rich global epigenomic profile from cells. Even in the presence of low-frequency heteroplasmic mutations, both methods successfully detected rare clones in clinical samples. However, these analyses require specific matching data types and are not applicable to single-cell RNA-sequencing (scRNA-seq), which has a reasonable capacity to profile many cell types simultaneously.

LINEAGE (label-free identification of endogenous informative single-cell mitochondrial RNA mutation for lineage analysis) was developed to overcome the inconvenience of requiring matching data modalities. LINEAGE is a “low cross-entropy subspace” separation and consensus clustering-based analysis that aims to identify informative mitochondrial RNA variants in label-free scRNA-seq data as endogenous markers to infer cell lineage relationships (Lin et al. 2022). Since LINEAGE requires high sequencing depth and coverage to effectively perform variant calling, it was designed for full-length scRNA-seq data such as Smart-seq2 and not for the more scalable technologies with strand bias, for example, 10 × Genomics scRNA-seq.

The mitochondrial alteration enrichment and genome analysis toolkit (maegatk) supporting computational pipeline were established following the introduction of the MAESTER protocol for mitochondrial read enrichment. maegatk is a refinement over the mgatk pipeline, constructed specifically to address the implicit technical biases inherent in sequencing transcriptomic libraries. Nucleotide concordance is conducted to establish a high base quality using unique molecular identifiers (UMIs), and reads are assigned based on mean base quality scores. Through the UMI consensus technique, maegatk also reduces the integration of sequencing errors; moreover, maegatk includes an indel-calling FreeBayes feature.

MQuad (mixture modelling of mitochondrial mutations) opened a new avenue for leveraging somatic mtDNA mutations as natural genetic barcodes to infer cellular relationships due to its broad applicability to various data modalities (Kwok et al. 2022). MQuad is an open-source tool that provides a comprehensive workflow for clonal analysis of scRNA-seq data and is constructed to integrate with other tools, such as cellsnp-lite (X. Huang and Huang 2021) and vireoSNP (Y. Huang et al. 2019). Expressed allele in single-cell data is piled-up using cellsnp-lite efficiently, and subsequently, MQuad can call clonally informative mtDNA variants in a population of single cells from single-cell RNA, DNA or ATAC sequencing data. MQuad depends on a binomial mixture model to determine mitochondrial heteroplasmy, which is directly adjusted as a proportional variable alongside raw read count instead of the conventional Gaussian mixture model. Given that sequencing data with high read counts often generate outputs with a higher proportion of false-positive variant discoveries, this enhancement is significantly relevant.

In this part, we briefly explored numerous existing tools that were recently created to perform variant calling and clonal inference in single-cell data. These tools are developed to tackle different challenges, mainly arising when analysing nuclear variants. Tools like Mutect2 (Benjamin et al. 2019), VarScan2 (Koboldt et al. 2012), and others are designed to detect low-frequency mutations with high sensitivity, distinguishing true mutations from sequencing errors and detecting low-frequency somatic mutations. They also address the influence of copy number variation on the detection of SNV status. The existence of indels introduces false SNVs during alignment. The Local Realignment Tool from GATK (Van der Auwera and O’Connor 2020) was designed to fix the alignment issues caused by indels.

When analysing mitochondrial mutation, new challenges need to be addressed. Mitochondrial DNA analysis is complicated by heteroplasmy (Stewart and Chinnery 2015b). The existence of mitochondrial drift (Campbell et al. 2023) and the mitochondrial DNA genetic bottleneck (Zhang et al. 2018) make both the detection of mitochondrial mutations and clonality inference more challenging. The interpretation of mutations is further complicated by their variable impact on cellular functions, particularly in diverse environments like tumour microecosystems (Kopinski et al. 2021). With the ever-improving state of sequencing technologies and introductions of novel sequencing protocols such as MAESTER, we expect continual advancements and evolutions to be required for the successful attempt to analyse the sequencing data generated and achieve significant biological inferences.

## Spatially resolved clonality analysis

**Fig. 3:**
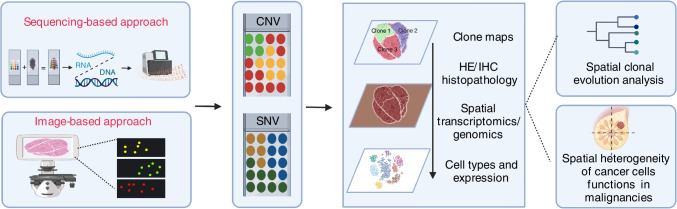
Spatial resolved clonal evolution. In sequencing-based approaches, spatial messenger RNA or targeted DNA profiling enable CNV inference and SNV calling for spatial resolved clonal evolution analysis. Imaging-based methods directly detect a panel of genomic alterations in situ to dissect spatial clonal heterogeneity

Single-cell analysis is a state-of-the-art technique that interrogates transcriptomics and genomics at an unprecedented resolution. This technique enables clonal evolution analysis at the single-cell level. However, conventional single-cell analysis is unable to preserve spatial information. Spatial transcriptomics is a field that emerged to study spatial heterogeneity of transcriptomics (Nagasawa et al. 2021) and was highlighted as the Nature Method of the Year 2020 (“Method of the Year 2020” 2021). It is difficult to achieve single-cell resolution for spatial transcriptomics, especially sequencing-based spatial transcriptomics. A compromised strategy for the sequencing-based approach is segregating cells from a tissue section into spots by either bead array (Boyd et al. 2020, p. 4) or hashing with DNA oligo (Srivatsan et al. 2021). Each spot contains 3–30 cells. The barcoded beads with spatial information capture mRNA and/or DNA of cells in the corresponding spot, resulting in an average expression of all cells within the spot. Hashing of DNA oligo with spatial information into the nuclei within individual spots enables demultiplexing the spatial information of individual nuclei, achieving single-cell spatial analysis (Fig. 3). Another spatially resolved approach is using high-resolution imaging (Fig. 3). The image-based approaches are restricted by a prior defined panel of up to 300 molecules which is 10 times less than that of the sequencing-based method (Nagasawa et al. 2021).

A growing number of studies have leveraged spatially resolved genomic information to decipher cancer progression. Spatial transcriptomic analysis adapts the CNV inference algorithm. In a broad sense, CNV inference from spatial transcriptomics data characterizing the spatial distribution of malignant cells or the boundary of tumours is a robust spatial clonal evolution analysis (Ji et al. 2020). Although both normal and malignant cells with an unknown ratio can be present in the same spot, CNV inference from spatial transcriptomics is informative for clonality analysis in primary human cancers (Erickson et al. 2022), in addition to identifying spots with malignant cells. Spatial genomic profiling is a more precise method to interrogate spatial clonal heterogeneity compared with CNV inference from spatial transcriptomics. Slide-DNA-seq has emerged as an approach to capture spatially resolved DNA sequences (Zhao et al. 2022). This study found that genetic clones are distributed in distinct spatial regions and that spatially distinct genetic clones are transcriptionally different. On the other hand, imaging-based genomic profiling of informative mutations identified by whole genome sequencing has been leveraged to investigate spatial clonal architecture in primary and metastatic breast cancer (Lomakin et al. 2022). This work showed that both monoclonal and polyclonal expansion contribute to cancer development, but often multiple clones co-occurred in the same lobule of the primary tumour. Furthermore, different genetic clones at metastatic lymph display distinct immune microenvironments.

There is a growing number of new methods for spatial analysis (Vandereyken et al. 2023). Among these, sequencing-based techniques, which examine RNA or DNA sequences, including open chromatin, are capable of lineage tracing using endogenous markers. Spatial ATAC-seq is a method that combines ATAC-seq principles (Assay for Transposase-Accessible Chromatin using sequencing) with spatial transcriptomics to study chromatin accessibility and gene regulation in a spatially resolved manner (Deng et al. 2022, p. 2). This approach allows researchers to investigate spatial endogenous mutations in open chromatin regions. Furthermore, Mission Bio has developed an innovative technology that utilises targeted amplicon sequencing for single-cell DNA analysis, enabling a deeper understanding of genetic variations within individual cells (Sun et al. 2023). When combined with nuclear oligo hashing, which facilitates the demultiplexing of spatial information for individual cells, this approach holds great promise for single-cell spatial DNA profiling and advancing lineage tracing with endogenous genetic alteration. Currently, most sequencing-based spatial omics techniques have limited resolution, typically capturing multiple cells in one spot for analysis. Spatial enhanced resolution omics-sequencing (Stereo-seq) overcomes this limitation by combining DNA nanoball (DNB)-patterned arrays with in situ RNA capture, achieving spatial analysis at subcellular levels (Zhao et al. 2022). The unprecedented resolution of Stereo-seq not only enables lineage tracing at the cellular level but also allows for subcellular lineage tracing in organelles such as mitochondria and chloroplasts.

## Summary and perspectives

Traditionally, lineage tracing approaches can be largely defined as retrospective and prospective. Prospective lineage tracing describes following the progress and development of the cells forward in time. Methods often employed in prospective lineage tracing include fluorescent reporter genes, CRE-recombinas and CRISPR-Cas9 methods (Bowling et al. 2020; Kebschull and Zador 2018; L. Li et al. 2023; Quinn et al. 2021; Tian et al. 2021; VanHorn and Morris 2021; Weinreb et al. 2020; Yang et al. 2022). However, although methods such as CRISPR-Cas9 bring about versatility and precision in the investigation of developmental biology, they also face significant challenges in data interpretation and possible off-target incidents (Zafar et al. 2020). Therefore, in recent developments, somatic nuclear and mitochondrial mutations that naturally occur during development and disease have been identified with great potential for retrospective lineage tracing (Wagner and Klein 2020). Read out of these mutations makes it possible to build lineages retrospectively using them as endogenous markers. Retrospective lineage tracing expands the clonality analysis from model organisms to human normal and pathological tissues for investigation of cell relationships during development and tumour evolution. However, nuclear mutation-based lineage tracing heavily relies on the whole genome or exome single-cell sequencing and is therefore currently low throughput (in the number of cells) and costly. Although as endogenous markers, the mutation rate is expected to be high enough for clonality reconstruction, the nature of low mutation rates in certain tissues and experimental organisms hindered the broad application of nuclear mutations.

By contrast, mitochondrial mutations could be attractive markers to chart clonality due to the high mutation rate and high copy numbers of the mitochondrial genome. The biological differences between tissues and experimental organisms are typically the choice for clonality analysis. MtDNA lineage tracing also has limitations in tracking early embryo and parallel clonal dynamics; therefore, the combination of both nuclear and mitochondrial mutations can decipher the cell relationships deeper. The computational algorithms for informative variant calling in a single cell can still be further developed. InferCNV, the most widely used tool for nuclear mutation inference, can lead to false-positive results caused by cell type-specific expression profiles and the unequal gene coverage biased (Durante et al. 2020). Multiple mitochondrial variants calling tools are only tailored for specific types of data. MQuad can be applied to most existing single-cell data with sufficient sequencing depth and even coverage; however, it does not consider the strand-specific allele information which potentially filters out some low-quality variants (Kwok et al. 2022).

The computational improvements in the future to call informative variants are expected to more accurately reconstruct the full lineage tree of highly complex biological systems. Finally, spatial transcriptomics provides a spatial resolution of genetic lineage tracing, helping us fully understand many biological processes such as cell migration during differentiation. The real single-cell resolution of spatially resolved lineage trees inferred from genetic lineage experiments is also an exciting future development. It is undeniable that lineage tracing plays an important role in uncovering cellular developmental processes. However, the field is still plagued by technical limitations, including scalability and low specificity. There is a need for constant updates in computational methodologies with increased compatibility with data generated from novel protocols. As demonstrated, recent developments and advancements hold strong potential and promise to allow for deeper insights into cell fate determination and pathological progression mechanisms. It is through continuous innovation and cross-disciplinary collaboration that we expect to see great progress in the blossoming field of lineage tracing.

Understanding the origin, current state, and future fate of cells in physiological and pathological contexts is a challenging task in biomedical research. This is primarily due to the low frequency of somatic mutations and their sparse distribution across the genome making them challenging to detect and analyse. Researchers must carefully design experiments and choose appropriate methods to overcome these challenges and accurately trace the lineage and fate of cells. Advances in single-cell genomics, next-generation sequencing technologies, and computational analysis methods are continually improving our ability to study and understand cell lineage dynamics. However, it remains a complex and ongoing area of research with many technical and biological considerations to address.

## Data Availability

No datasets were generated or analysed during the current study.
